# In vitro co-culture of *Fasciola hepatica* newly excysted juveniles (NEJs) with 3D HepG2 spheroids permits novel investigation of host–parasite interactions

**DOI:** 10.1080/21505594.2025.2482159

**Published:** 2025-03-25

**Authors:** Aiste Vitkauskaite, Emma McDermott, Richard Lalor, Carolina De Marco Verissimo, Mahshid H. Dehkordi, Kerry Thompson, Peter Owens, Howard Oliver Fearnhead, John Pius Dalton, Nichola Eliza Davies Calvani

**Affiliations:** a Molecular Parasitology Laboratory, Centre for One Health, Ryan Institute, School of Natural Sciences, The University of Galway, Galway, The Republic of Ireland; b Anatomy Imaging and Microscopy (AIM), Anatomy, School of Medicine, The University of Galway, Galway, The Republic of Ireland; c Pharmacology and Therapeutics, School of Medicine, The University of Galway, Galway, The Republic of Ireland

**Keywords:** *Fasciola hepatica*, liver fluke, HepG2, spheroids, 3D cell culture, helminth

## Abstract

*Fasciola hepatica*, or liver fluke, causes fasciolosis in humans and livestock. Following ingestion of vegetation contaminated with encysted parasites, metacercariae, newly excysted juveniles (NEJ) excyst in the small intestine and cross the intestinal wall. After penetrating the liver, the parasite begins an intra-parenchymal migratory and feeding phase that not only drives their rapid growth and development but also causes extensive haemorrhaging and immune pathology. Studies on infection are hindered by the difficulty in accessing these microscopic juvenile parasites *in vivo*. Thus, a simple and scalable *in vitro* culture system for parasite development is needed. Here, we find that two-dimensional (2D) culture systems using cell monolayers support NEJ growth to a limited extent. By contrast, co-culture of *F. hepatica* NEJ with HepG2-derived 3D spheroids, or “mini-livers,” that more closely mimic the physiology and microenvironment of *in vivo* liver tissue, promoted NEJ survival, growth, and development. NEJ grazed on the peripheral cells of the spheroids, and they released temporally regulated digestive cysteine proteases, FhCL3, and FhCL1/2, similar to *in vivo* parasites. The 3D co-culture induced development of the NEJ gut and body musculature, and stimulated the tegument to elaborate spines and a variety of surface sensory/tango/chemoreceptor papillae (termed S1, S2, and S3); these were especially pronounced around the oral and ventral suckers that sense host chemical cues and secure the parasite in tissue. HepG2 3D spheroid/parasite co-culture methodologies should accelerate investigations into the understanding of *F. hepatica* NEJ developmental biology and studies on host–parasite interactions, and streamline the search for new anti-parasite interventions.

## GRAPHICAL ABSTRACT

**Figure:**
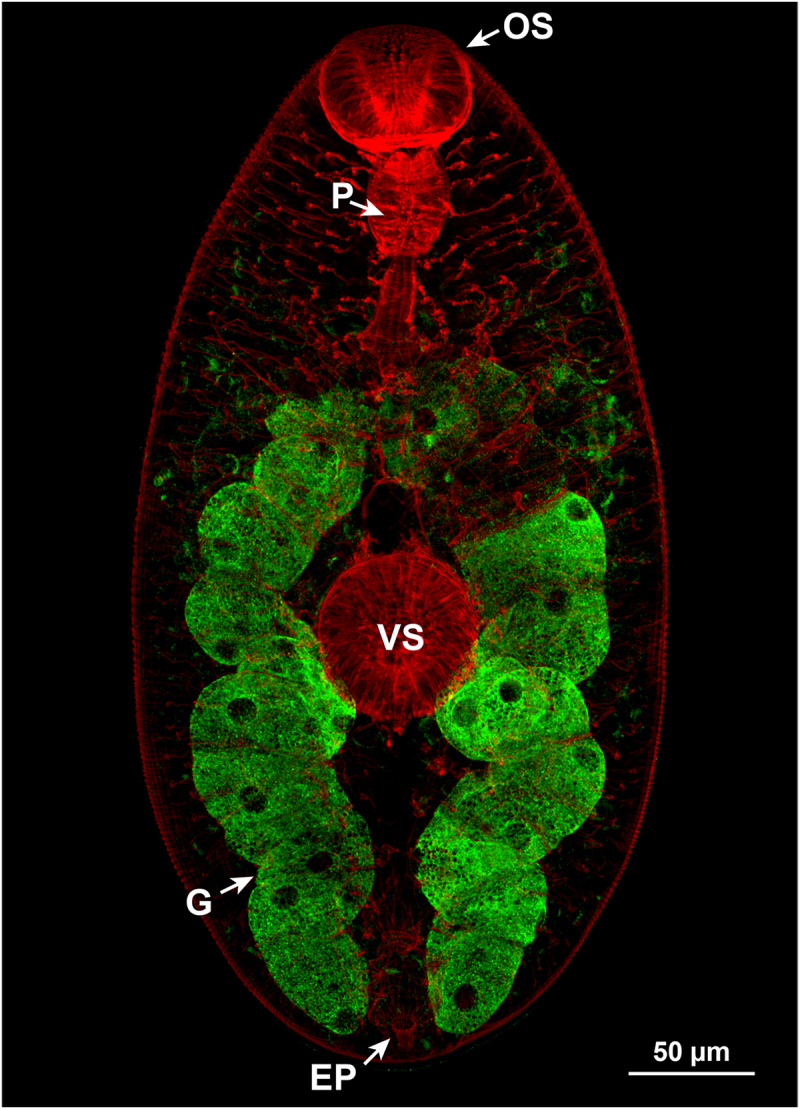

## Introduction

Helminth (worm) pathogens cause chronic disease in more than two billion people globally [1,2]. These multicellular parasites undergo complex growth and developmental changes in their mammalian hosts that are often complimented with convoluted migratory paths through various host tissues. Most studies on helminths focus on parasites that can be readily maintained in the laboratory using *in vivo* models, usually rodents, and on the more readily accessible stages, such as adults [3]. *In vitro* culture systems have proven invaluable in supporting molecular investigations of parasite somatic and secreted molecules and extracellular vesicles (EVs) that are important for infection, migration, and host interaction. However, in most cases, they fail to support the long-term growth and developmental needs of the parasites, hindering the advancement of simple high-throughput methods for the discovery of anti-parasitic drugs and vaccines [3]. The development of 3D cell culture systems involving multi-cellular tissue constructs, organoids, has advanced credible *in vitro* host–pathogen interaction studies. The establishment of ruminant and murine-derived gastrointestinal organoids has revealed important details about the invasion and colonisation mechanisms of several major gastrointestinal nematodes (GIN) including *Teladorsagia circumcincta, Ostertagia ostertagi, Haemonchus contortus*, and *Trichuris muris* [4–10].

Alternative *in vitro* systems for growing cells *in vitro* in 3D, rather than 2D, are commonly employed in fields such as tumour biology and neuroscience because they are considered to better mimic the physiology and microenvironment of solid tissues [11–13]. Spheroidal masses of homotypic cells, termed spheroids, have the potential to simulate various host tissues *in vitro*, and have demonstrated increased suitability in the field of toxicology and drug screening compared to 2D cell cultures [11–16]. As a consequence, their application as *in vitro* pre-screening systems prior to studies advancing to animal models, and their compliance with the 3 R principles to reduce, replace, and refine *in vivo* models has seen their use increase in recent years [11,12,15,17]. However, 3D spheroidal cell culture systems have yet to be explored as a solid tissue model to investigate parasite developmental biology and host–parasite interactions.

The flatworm *Fasciola hepatica* causes fasciolosis, a zoonosis that afflicts over 17 million people and is a scourge of livestock production across the globe [18–22]. Infection of the mammalian host occurs following consumption of aquatic vegetation contaminated with the encysted infective-stage parasite, or metacercariae. Once in the duodenum, newly excysted juveniles (NEJ) emerge from their cysts and within hours traverse the intestinal wall. Over the subsequent week, they migrate *via* the abdominal cavity into the liver parenchyma where their extensive feeding and tunnelling activity for the next eight to 10 weeks causes severe bleeding, haemorrhaging, and immune-mediated pathology; within this time parasites grow exponentially from the microscopic size of ~100 µm to over 1 cm in length [21,23,24]. Clinical impacts of fasciolosis during this acute-stage of infection range from severe liver damage to sudden death in animals, and later on to a chronic debilitating infection of the liver and bile ducts with associated decreases in animal fertility, meat, milk, and wool production [19,20,23,25–30]. Future interventions, such as new vaccines or drugs, need to focus on preventing NEJ from penetrating and accessing the liver [31–34]. However, there has been a dearth of biological investigations on these early parasite stages because they are extremely difficult to recover from *in vivo* model hosts (mice, rats).

Here, we established an *in vitro* growth and development model for *F. hepatica* NEJ using commercially available cell lines suitable for 3D cell culture. We selected the HepG2 liver cell line to develop a procedure for producing spheroids, or “mini-livers,” of optimal age and size to support *F. hepatica* NEJ growth and development *in vitro* for over 3 weeks. We used biochemical and advanced microscopic methods, including live cell imaging, to reveal novel insights into the growth dynamics and behaviour of these early invasive pathogen stages. In addition, we show, for the first time, how the physical interaction of NEJ with the microtissue results in the development and expression of tegumental spines and several types of sensory structures distributed on their surface, particularly on and adjacent to their oral and ventral suckers. This new *in vitro* parasite–host interaction model allows the study of *F. hepatica* growth and development at the stages not readily accessible *in vivo*. Alongside expediting our search for new vaccines and anti-parasitics, this model serves as an example for the development of similar systems for other economically important zoonotic helminths.

## Materials and methods

### HepG2 cell culture and spheroid production

Human hepatoma HepG2 cells were obtained on two occasions from the same supplier (85011430, Sigma-Aldrich). Following thawing, these were cultured and passaged in a filter sterilised by Dulbecco’s Modified Eagle Medium (DMEM; high glucose, glutamax supplement 61,965,026, Gibco, UK) containing 10% (*v/v*) foetal bovine serum (FBS; 10500064, Gibco) and 1% (*v/v*) Antibiotic-Antimycotic (15240062, Gibco) to 70–80% confluence. Cells were cultured for two to five passages prior to harvesting using Trypsin-EDTA (2× concentration, T4174, Sigma-Aldrich).

Two-dimensional (2D) adherent cell cultures were generated by seeding 1,000, 1,500, 2,000, 3,000, and 4,000 cells/well into flat-bottom 96 well sterile tissue culture microplates in the same medium as described above (83.3924, Sarstedt). After seeding, the plates were incubated for 24 h at 37 °C in 5% CO_2_ to allow cell attachment [17]. Then, five NEJs were pipetted into each well and daily observations began.

Individual spheroids were generated by seeding 200 cells/well in ultra-low attachment (ULA) U-bottom 96 well cell culture microplates (174925, Thermo Scientific) containing 200 μL of complete DMEM. The plates were then centrifuged at 400 × *g* for 10 min to encourage cell aggregation and spheroid formation. Spheroids were maintained by replacing the medium 1:1 three times per week and centrifuging the plates at 260 × *g* (MPW 380 R centrifuge, MPW Med. Instruments, Warsaw, Poland). Spheroids were incubated at 37 °C in 5% CO_2_ for 13 days prior to parasite co-culture [17]. On the twelfth day of culture, spheroids were plated in triplet (3 spheroids/well) and incubated overnight to allow the spheroids to merge.

Spheroids and 2D monolayer cell cultures were imaged using phase contrast microscopy (Nikon eclipse TSD100 inverted microscope, Nikon Corporation, Tokyo, Japan) every second day using Euromex ImageFocus Alpha software v1.3.7 (Euromex Microscopen bv, Arnhem, the Netherlands).

### Excystment of *F. hepatica* NEJ and HepG2 co-culture

*F. hepatica* metacercariae (Italian isolate; Ridgeway Research, UK) were excysted as previously described [23,34]. Briefly, the outer cyst walls were removed by agitation in 2% sodium hypochlorite solution for a maximum of 8 min. Metacercariae were washed three times in deionised water prior to incubation in the excystment medium: (1.2% (*w/v*) sodium bicarbonate, 0.9% (*w/v*) sodium chloride, 0.2% (*w/v*) sodium tauroglycocholate, 0.07% (*v/v*) concentrated hydrochloric acid, 0.006% (*w/v*) L-cysteine, all from Sigma-Aldrich) for 2 h at 37 °C in 5% CO_2_. Upon excystment, the NEJ were washed three times in a filter sterilised with DMEM containing 10% (v/v) FBS and 1% (v/v) Antibiotic-Antimycotic, before co-culture with 2D adherent cells and 13-day-old spheroids.

**Figure 1.**
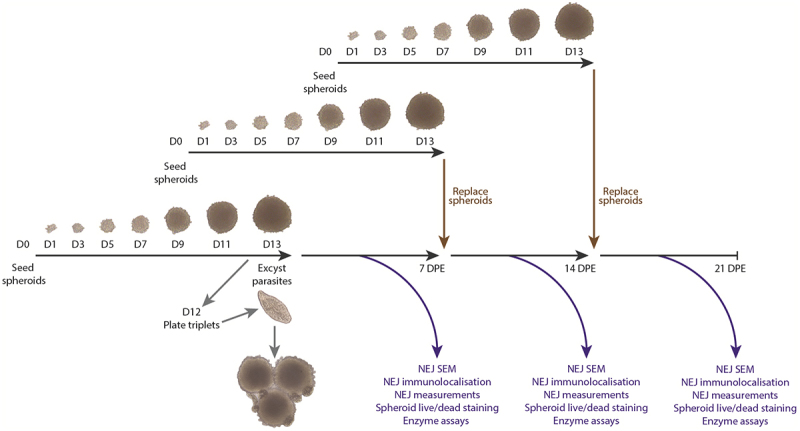
*Fasciola hepatica*-HepG2 spheroid co-culture workflow. Spheroids derived from HepG2 cells were seeded (200 cells/well) in an ultra-low attachment (ULA) U-bottom 96 well cell culture microplates containing 200 μL of complete DMEM and grown in individual wells for 12 days. On day 13, three spheroids were co-cultured with five *F. hepatica* NEJ. On day seven, 14 and 21 DPE, parasites were removed from the wells and either plated with fresh 13-day spheroids or fixed in fixatives for analysis by scanning electron microscopy (SEM), immunolocalisation, and length/width measurement. At the same timepoints, aliquots of cell culture media from each treatment group (media alone, NEJ without spheroids, spheroids alone, NEJ with spheroids) were collected and analysed for enzyme activity assays. The same workflow was followed for the co-culture with HepG2 2D adherent cells.

Five NEJ/well were co-cultured with and without 2D adherent cells or triplet spheroids in the ULA U-bottomed 96 well plates (across two independent experiments; 10 wells per treatment group per each experiment) for up to 21 days at 37 °C in 5% CO_2_. Triplet spheroids and 2D adherent cells were cultured without NEJ as negative controls. Half of the cell culture medium was replaced three times per week, and the parasites were transferred to a new plate with fresh 2D adherent cells or 13-day-old spheroids every 7 days (Figure 1).

Parasite viability was assessed each day for survival rate calculations. Parasites were considered dead if they lacked movement and appeared heavily granulated. Immature parasites were fixed at 7, 14, and 21 days post-excystment (DPE) as described below. At the time of fixation, parasite length and width from a random subset of 10 parasites/treatment/week were measured using the Euromex ImageFocus Alpha software v1.3.7 on a Nikon eclipse TSD100 inverted light microscope (Nikon Corporation, Tokyo, Japan) equipped with a CMEX-18PRO USB camera (Euromex Microscopen bv, Arnhem, the Netherlands).

### Immuno-detection of cysteine protease expression in NEJ

NEJ (0-h post excystment; HPE) were fixed in 4% paraformaldehyde (PFA; Sigma-Aldrich) in 0.1 M PBS (Sigma-Aldrich) pH 7.4, for 1 h at room temperature (RT). Immature parasites (7, 14, and 21 DPE) were fixed for 4 h at RT. After three washes in the antibody diluent buffer (AbD buffer; PBS containing 0.1% (v/v) Triton X-100, 0.1% (w/v) bovine serum albumin, and 0.1% (w/v) sodium azide, all from Sigma-Aldrich), the parasites were incubated in either rabbit pre-immune sera or polyclonal antibodies prepared against recombinant *F. hepatica* cysteine proteases, namely cathepsin L3 (anti-rFhCL3) and cathepsin L1 propeptide (anti-pprFhCL1), diluted 1:500 in AbD buffer overnight at 4°C. After three washes in AbD, the parasites were incubated in a 1:200 dilution of the secondary antibody, fluorescein isothiocyanate (FITC)-labelled goat-anti-rabbit IgG (Sigma-Aldrich) overnight at 4 °C in the dark. To counter-stain muscle tissue, the samples were incubated in an AbD buffer containing phalloidin-tetramethylrhodamine isothiocyanate (TRITC) (200 μg/mL) overnight in the dark at 4 °C. The parasites were washed three times in an AbD buffer, whole-mounted onto slides using 10% glycerol solution containing 0.1 M propyl gallate and visualised under an Olympus Fluoview 3000 Laser Scanning confocal microscope using a 60× UPLANSAPO oil immersion lens. Olympus type F immersion oil was used in viewing, and all images were taken at room temperature [34,35].

### Scanning electron microscopy (SEM)

NEJ obtained after excystment and juvenile parasites at 7, 14, and 21 DPE were fixed in 2% glutaraldehyde and 2% PFA in 0.1 M sodium cacodylate buffer pH 7.2 overnight at 4 °C. The samples were dehydrated through a graded series of ethanol (30%, 50%, 70%, 90%, and 100%) for 2 × 15 min per concentration. The samples were critical point dried (Leica EM CPD300), sputter-coated with gold (Quorum Q150R ES Plus) and imaged using a Hitachi S-4700 scanning electron microscope.

### Time lapse live cell imaging

To capture early parasite–spheroid interactions, individual 12-day-old spheroids were plated in triplet on 96 well clear round bottom ultra-low attachment black microplates (4520, Corning) and incubated overnight at 37 °C in 5% CO_2_ to allow the spheroids to merge. The following day, 0 hPE NEJ were added to wells with and without spheroids. Wells were imaged once every 30 sec for 13 h.

To assess whether parasites ingest cells, while incubated with spheroids, individual 12-day-old spheroids were stained with PKH67 green fluorescent membrane dye as per the manufacturer’s instructions (MIDI67-1KT, Sigma-Aldrich). Spheroids were plated in triplet on 96 well clear round bottom ULA black microplates (4520, Corning) and incubated overnight at 37 °C in 5% CO_2_ prior to co-incubation with parasites for up to 7 days. Live imaging of parasites was carried out using a FV3000 Olympus Fluoview 3000 Laser Scanning Inverted confocal microscope equipped with a 10× UPLANSAPO objective lens (imaged every 60 sec for 21 h). Prior to the start of the live imaging experiments, the microscope and environmental chamber were heated to 37 °C to minimise thermal and mechanical drifts. The humidified gas was maintained at 5% CO_2_ over the duration of the time course.

### Spheroid staining and imaging

13-day and 20-day spheroids cultured with or without parasites were transferred to 96 well clear round bottom ultra-low attachment black microplates (4520, Corning). Spheroids were stained with calcein AM (live cells) and BOBO-3 Iodide (dead cell nuclei) using a commercially available LIVE/DEAD® cell imaging kit (488/570) (R37601, Invitrogen) as per the manufacturer’s instructions. Spheroids were additionally stained with Hoechst 33,342 (H3570, Invitrogen, final concentration 1 µg/mL) (both live and dead cell nuclei). Spheroids were stained at 37 °C in 5% CO_2_ for 1 hour prior to imaging. Imaging was carried out using an automated confocal microscope on the Perkin Elmer Operetta high-content imaging system (Perkin Elmer, Waltham, MA, USA) [17].

### Determination of FhCL3 and FhCL1/2 enzymatic activity in culture medium

Native cysteine proteases released by *F. hepatica* parasites into culture media when incubated with and without spheroid co-culture were characterised by enzymatic activity using a fluorogenic substrate assay [36–39]. The samples, aliquots of cell culture supernatants at 7, 14, and 21 DPE were collected from wells containing media alone (negative control), spheroids only, and parasites with and without spheroids and stored at −20 °C throughout the experiments (a minimum of five wells were pooled). On the day of analysis, the samples were thawed on ice and pre-incubated 1:1 (*v/v*) with 100 mm sodium acetate buffer, pH 5.5, containing 1 mm DTT, 1 mm EDTA, and 0.01% Brij L23 for 10 min at 37 °C at a final volume of 50 µL. The reaction volume was then brought up to 100 µL by the addition of the fluorogenic substrates dissolved in the same sodium acetate buffer. The specific activity of *F. hepatica* cathepsin L3 (FhCL3) or cathepsin L1/L2 (FhCL1/2) within the supernatants was assessed with Z-Gly-Pro-Arg-NHMec (20 µM, Bachem) and Z-Leu-Arg-NHMec (20 µM, Bachem), respectively, for up to 1 h at 37 °C, as relative fluorescent units (RFU) on a PolarStar Omega Spectrophotometer (BMG LabTech). The broad-spectrum cysteine protease inhibitor E-64 (100 µM; Sigma Aldrich) was used to ensure the activity observed was derived from cysteine proteases. Active recombinant enzymes (rFhCL3 and rFhCL1) served as positive controls for the assays. All reactions were carried out in triplicate.

### Statistical analysis

Data was collected, stored, and analysed in Microsoft Excel version 16 and GraphPad Prism for MacOS version 10.0.3. Differences in parasite size when cultured without cells and with varied adherent cell densities were assessed using an ordinary one-way ANOVA with Tukey’s multiple-comparison test. Parasite survival data when cultured with and without adherent cells or spheroids were analysed by a log-rank (Mantel-Cox) test. Statistical significance in parasite growth in 3D spheroid co-culture was assessed using a two-way ANOVA followed by Tukey’s multiple-comparison test. Differences in FhCL3 or FhCL1/2 enzymatic activity within culture media supernatants collected during parasite co-culture with and without spheroids/2D adherent cells were assessed using a two-way repeated measures ANOVA followed by Tukey’s multiple comparison test. *p* values of ≤ 0.05 were considered significant. Significant differences between the groups are indicated by asterisk(s): *p ≤ 0.05, **p ≤ 0.01, ***p ≤ 0.001, ****p ≤ 0.0001.

## Results

### HepG2 cell co-culture supports *F. hepatica* growth in vitro

**Figure 2.**
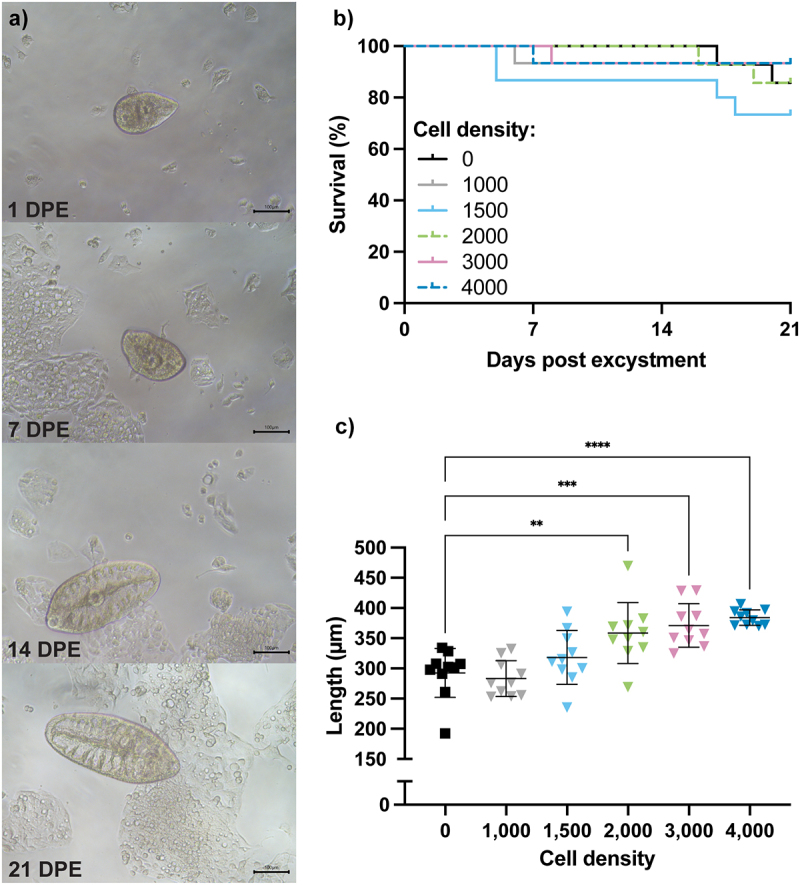
Co-culture of *F. hepatica* NEJ with adherent (2D) HepG2 cells increases parasite growth in a cell density-dependent manner. (a) In 2D HepG2 cell cultures, *F. hepatica* NEJ were observed regularly interacting with clumps of adherent cells. Photographs shown were taken from cultures with 4,000 cells per well (see B). Scale bar: 100 µm. (b) NEJ survival was >90% when cultured in the presence of the monolayer of cells at various densities (*p* > 0.05, log-rank (Mantel-Cox) test, *N* = 14–15 per group). (c) Parasite length increased with increasing cell seeding density, with a significant difference observed between parasites cultured without cells and those cultured with a minimum of 2,000 cells seeded per well at 21 DPE (**p < 0.01, ***p < 0.001,****p < 0.0001, ordinary one-way ANOVA with Tukey’s *post hoc* test, *N* = 10 per group). DPE, days post excystment.

In our initial *in vitro* experiments, we maintained *F. hepatica* NEJ with adherent 2D cultures of HepG2 cells containing 10% FBS for up to 21 days (DPE) (Figure 2(a)). While parasite survival in the presence of HepG2 cells was comparable to those cultured in medium without cells (Figure 2(b)), parasites cultured with HepG2 cells exhibited a significant increase in size as assess by parasite length (p < 0.01). In addition, we found that with higher cell densities, the increase in parasite growth was greater (Figure 2(a,c); Supplementary Data S1). Notably, however, the parasites interacted more regularly with areas of the culture where the HepG2 cells clumped into small concentrated masses over the 21-day culture period (Figure 2(a)); the parasites orientated their oral sucker towards the cell clumps, pushing towards them and had regular peristaltic movements of the gut while ingesting and feeding on the cells (see Figure 2(a) and Supplementary Video 1). These 2D cell culture studies suggested that the HepG2 cells provide growth-stimulating factors for the parasite, a source of nutrients, as well as a substrate with which the parasite could physically interact. These observations prompted us to explore the suitability of more advanced 3D cell cultures in the form of HepG2-derived spheroids as an *in vitro* model to investigate NEJ invasion, survival, growth, and development.

### Co-culture with spheroids increases parasite viability and survival

HepG2 spheroids were prepared using protocols previously developed in our laboratory [17] which established an optimal seeding density of 200 hepG2 cells/well for their preparation. However, to create a practical protocol for investigating parasite–spheroid interaction, we cultured different numbers of NEJ with various numbers of spheroids that ranged in age/size from 7 to 13 days old. By assessing both parasite and spheroid growth, and monitoring parasite viability and locomotion (at least three replicates), testing at least three batches of HepG2 cells, we characterised the success of the cultures and arrived at a method that was compatible with both parasite and spheroid growth. The final established protocol involved culturing five NEJ with three 13-day-old spheroids per well, with the culture medium changed every 2 days and spheroids replaced every 7 days with fresh 13-day-old spheroids. This protocol is presented schematically in Figure 1.

**Figure 3.**
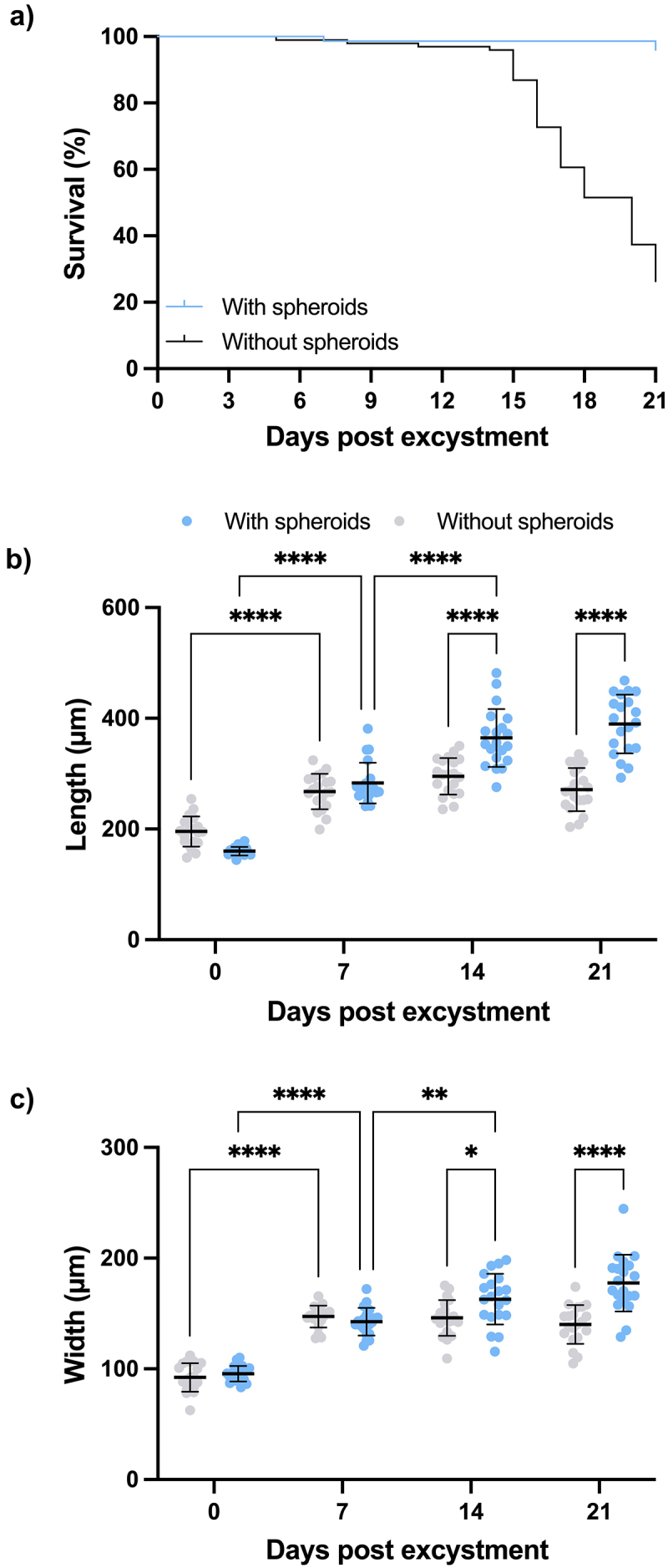
Co-culture with HepG2 spheroids promotes *F. hepatica* NEJ survival and growth *in vitro*. (a) *F. hepatica* NEJ survival over 21 days was significantly improved when cultured with HepG2 spheroids compared to those without spheroids (*p* < 0.0001, log-rank (Mantel-Cox) test; with spheroids: *N* = 148, without spheroids: *N* = 99 across two independent experiments). Change in length (b) and width (c) of *F. hepatica* NEJ cultured without (grey circles) or with (blue circles) HepG2 spheroids in two independent experiments over 21 days. Asterisks indicate significant differences between the two groups (NEJ cultured without vs with spheroids) at day 0, 7, 14 and 21, and differences between different timepoints within the same group (NEJ without or with spheroids). *p < 0.05, **p < 0.01, ****p < 0.0001, two-way ANOVA with Tukey’s *post hoc* test, *N* = 20 per group across two independent experiments.

**Figure 4.**
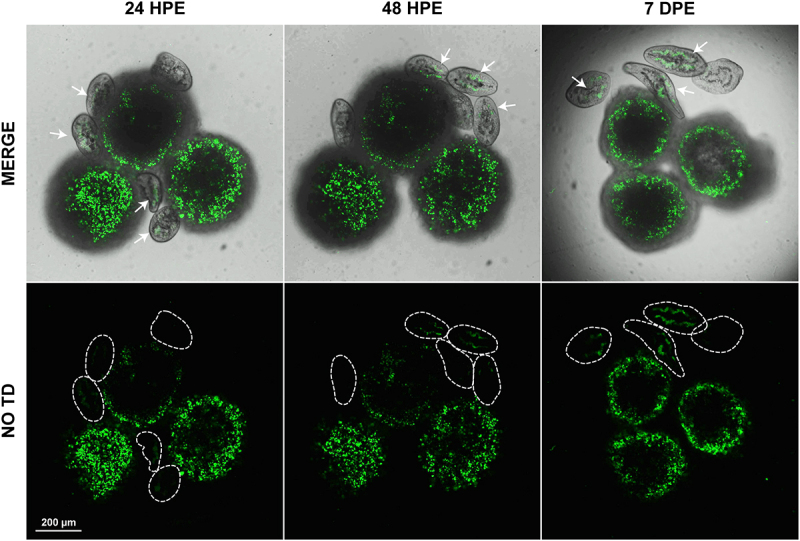
Parasites graze on and ingest cells derived from HepG2 spheroids during co-culture. Spheroids stained with PKH67 green fluorescent membrane dye were incubated with *F. hepatica* NEJ for 7 days. Green fluorescence was evident in parasite guts within the first 24 h post-excystment (HPE) and remained pronounced until seven days post excystment (DPE). Top row of images: an overlay of the transmitted light (TD) and Alexa Fluor 488 (green) fluorescent channel; bottom row: the fluorescent channel only. Scale bar: 200 μm.

By employing the above protocol we could readily maintain parasites with spheroids for up to 21 days. Parasite survival over this period was significantly enhanced by co-culture with spheroids compared to parasites cultured in the medium alone (*p* < 0.0001) (Figure 3(a); Supplementary Data S2). This new system of co-culture allowed us to examine NEJ interactions and behaviour with spheroids, which represent “mini-livers” and mimic *in vivo* parasite–host interactions. Indeed, using real-time live cell microscopy over 13 h we observed significant differences in the activity and motility between NEJ cultured with spheroids and those cultured alone (Supplementary Video 2 and 3). As shown in the videos presented (see supplementary Video 3), it is clear that parasites co-cultured with spheroids were highly mobile and active, constantly moving around the edges of the spheroids and sometimes moving a distance away and returning. They were also seen making close tangential contact to the spheroids, and frequently penetrating/invading the spaces between the spheroids with a forward movement (sufficiently forceful as to move the spheroids). In addition, the NEJ appeared to be “grazing” on the spheroids, and regular contraction and expansion movements of the gut and its contents were clearly visible (Figure 4; Supplementary Video 4). After a week of co-culture with parasites, spheroids became visibly smaller and their surface uneven indicating that the parasites had fed on the spheroids, removing and ingesting cells from the periphery. By comparison, spheroids cultured without parasites did not lose the overall spherical shape, increased in size, and maintained a smooth and intact surface (Supplementary Figure S1).

In stark contrast, juvenile parasites cultured in the medium without spheroids became progressively dormant over the 21-day study period and exhibited a decline in overall body movement and in gut activity beginning from approximately 7 DPE. In addition, the survival of the parasites cultured without spheroids rapidly declined from 14 DPE and most had died by 21 DPE (Figure 3(a)).

### Parasites graze on and ingest the peripheral cells of spheroids

We used the green fluorescent lipid-soluble dye PKH67 to stain the spheroid membranes and determine if cells were ingested and subsequently digested by the growing parasites. Green-stained spheroids were prepared and incubated with the NEJ and examined using live cell microscopy. The uptake of green spheroid-derived cells into the parasite gut was observed within 24 hPE and could still be observed at 7 DPE (Figure 4; Supplementary Video 4). Because there is limited transfer of the PKH67 dye to daughter cells within the spheroids, unstained cells gradually accumulate on the surface of the spheroids over the 7 days of parasite co-culture. Hence, the number of non-fluorescently-labelled cells ingested into the parasite guts gradually increases each seven-day culture period.

### Co-culture with HepG2 spheroids promotes F. hepatica growth and development

Parasites co-cultured with HepG2 spheroids were significantly larger (length: *p* < 0.0001 on both 14 and 21 DPE, width: *p* = 0.0328 on 14 DPE and *p* < 0.0001 on 21 DPE) than parasites cultured in medium alone (Figure 3(b,c); Supplementary Data S2). Parasites in spheroid co-culture exhibited a significant increase in size from 0 DPE to 14 DPE (length: *p* < 0.0001 on both 7 and 14 DPE, width: *p* < 0.0001 on 7 DPE, *p* = 0.0038 on 14 DPE) at which timepoint, they reached their growth capacity with neither parasite length nor width showing a statistically significant increase between 14 and 21 DPE. By contrast, parasites cultured without spheroids reached their growth limit at 7 DPE (Figure 3(b,c); Supplementary Data 2).

**Figure 5.**
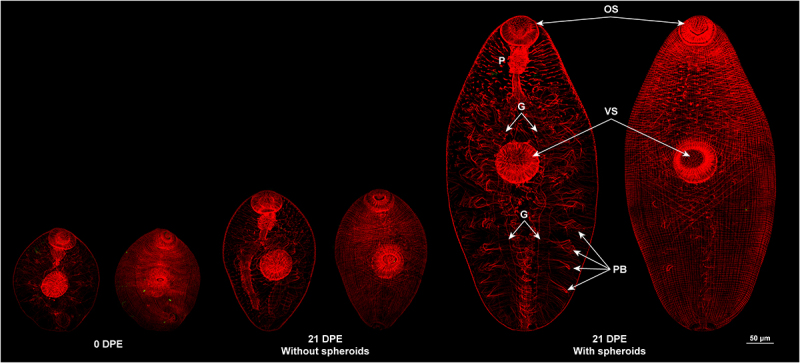
Co-culture with HepG2 spheroids increases parasite size, gut complexity and muscle development. Representative *F. hepatica* NEJ were fixed at excystment (0 days post-excystment – DPE) and 21 DPE after co-culture with and without HepG2 spheroids. Phalloidin-tritc was used to stain muscle tissue (actin: red fluorescence) before parasites were whole-mounted for laser-scanning confocal microscopy. Parasites on the left display internal musculature, while parasites on the right show surface musculature. OS: oral sucker; P: pharynx; G: two main caeca of the gut; VS: ventral sucker; PB: primary branching of the digestive caeca. Scale bar: 50 μm.

In addition, parasites cultured with spheroids show a clear development of the gut caeca compared to parasites grown without spheroids, evidenced by increased muscle development around the bifurcated gut, which was visualised by actin staining with Phalloidin-TRITC (Figure 5). The total body musculature, that surrounding the gut caecum, and particularly the musculature of oral and ventral suckers in parasites at 21 DPE took on a mature adult-like morphology. On the other hand, parasites that were cultured for 21 DPE without spheroids displayed atrophy of the gut musculature and sub-tegumental musculature, and retained an immature-like morphology. The parasites co-cultured with spheroids were much larger in size compared to parasites cultured without spheroids at 21 DPE (Figure 5).

**Figure 6.**
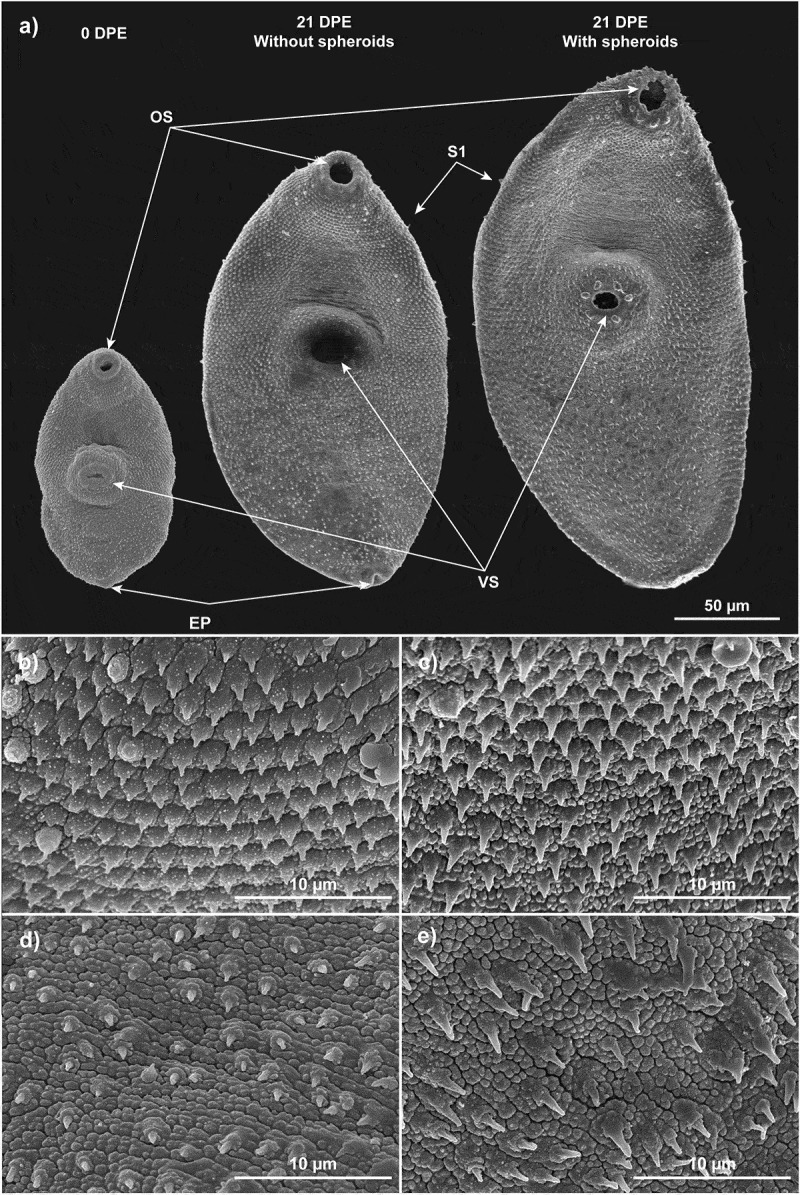
Co-culture with HepG2 spheroids spurs *F. hepatica* NEJ growth and development of tegument structure and organisation. (a) Scanning electron microscopy (SEM) demonstrating the external anatomy of *F. hepatica*NEJ post-excystment (0 DPE) compared with parasites co-cultured with and without HepG2 spheroids for 21 DPE. Scale bar: 50 μm. (b) SEM of the collar of *F. hepatica* 21 DPE cultured in media without spheroids shows limited spine growth and reduced surface texture. Scale bar: 10 μm. (c) Co-culture with HepG2 spheroids for 21 days promotes spine development and surface complexity as demonstrated by SEM of the tegument below the oral sucker. Scale bar: 10 μm. (d) SEM of the ventral distal spines of *F. hepatica* at 21 DPE show limited development in the absence of HepG2 spheroids. Scale bar: 10 μm. (e) SEM of the ventral distal surface of *F. hepatica* 21 DPE co-cultured with HepG2 spheroids shows increased spine length and aberrant organisation typical of parasites grown *in vivo*. Scale bar: 10 μm. OS: Oral sucker; VS: Ventral sucker; S1: Ciliated sensory papillae; EP: Excretory pore.

Scanning electron microscopy (SEM) was previously employed by Bennett and colleagues [40,41] to visualise microscopic changes that occur on the surface tegument during the early growth of *F. hepatica in vivo*, as this is the parasite tissue in close contact with the host tissue and involved in a continuous molecular interplay. In the present study using SEM, we could distinguish significant differences in the surface ultrastructure and morphology of parasites co-cultured with HepG2 spheroids compared to those cultured without spheroids (Figure 6(a)). The most conspicuous structures on the parasite surface are single-pointed spines which are considered essential in aiding parasite migration through the liver during early infection; these are less developed in parasites cultured without spheroids (Figure 6(b,d)) than in those co-cultured with spheroids (Figure 6(c,e)). Spine length and organisation are also altered by interaction with spheroids, with parasites grown in co-culture displaying longer spines compared to those cultured in media alone (Figure 6(b–e)). As described for parasites obtained *in vivo* [40,42], the oral sucker is free of spines. Below this structure, spines form a collar of regular rings and point backwards, while those below the ventral suckers are less dense and more irregular. Spines are missing from the area immediately above the ventral sucker where the genital pore will eventually open [40,42].

**Figure 7.**
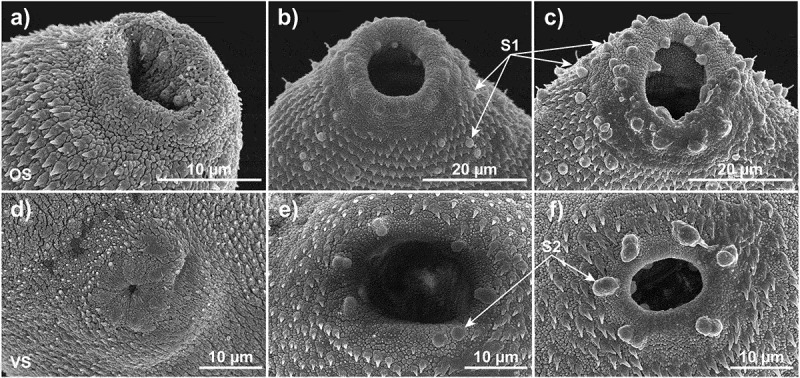
Interaction with HepG2 spheroids stimulates the development of *F. hepatica* NEJ external anatomy, including sensory organs. (a) The oral sucker of *F. hepatica* NEJ immediately post excystment shows primordial sensory organs in and around the opening of the oral sucker. Scale bar: 10 μm. (b) *F. hepatica* cultured without spheroids for 21 days have underdevelopment of ciliated sensory papillae (S1) around the oral sucker. Scale bar: 20 μm. (c) The sensory organs (ciliated sensory papillae, S1, and domed sensory papillae, S2) around the oral sucker of *F. hepatica* NEJ after 21 days of co-culture with HepG2 spheroids are very pronounced, both in terms of size and number. Scale bar: 20 μm. (d) Sensory organs are underdeveloped around the ventral sucker of the NEJ at excystment. Scale bar: 10 μm. (e) The spines and domed sensory papillae (S2) around the ventral sucker of *F. hepatica* after 21 days of culture without HepG2 spheroids show limited development. Scale bar: 10 μm. (f) The spines and domed S2 sensory papillae are prominent around the ventral sucker *F. hepatica* NEJ after 21 days of co-culture with HepG2 spheroids. Scale bar: 10 μm. OS: Oral sucker, VS: Ventral sucker; S1: Ciliated sensory papillae; S2: Domed sensory papillae.

**Figure 8.**
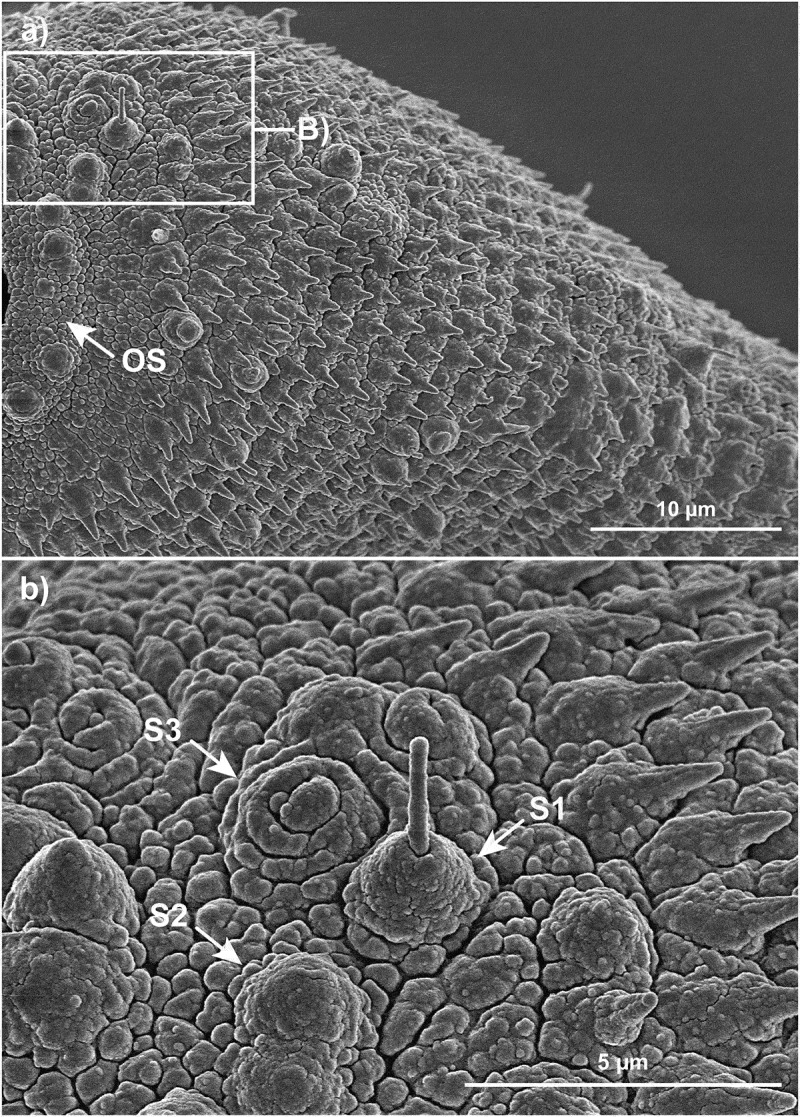
The different forms of sensory papillae. (a) The distribution of spines and sensory papillae adjacent to the oral sucker of *F. hepatica* after 21 days of culture with HepG2 spheroids. Scale bar: 10 μm. (b) Close-up of the three different sensory papillae highlighted within a box in A; the sensory papillae are named after Bennett (1975) as ciliated (S1), domed (S2) and shielded (S3). Scale bar: 5 μm.

Sensory papillae are a particularly conspicuous developmental feature on the parasite tegument. Our SEM studies agree with Bennett [40] and show that these sensory structures are underdeveloped in the NEJ (Figure 7(a–d)) and remained underdeveloped in parasites that were cultured without spheroids (Figure 7(b–e)). However, these structures become very obvious, as early as 7 DPE, on the tegument of parasites that are cultured with spheroids (Figure 7(c,f)), particularly in the anterior end of the parasite around the inner and outer rims of the oral sucker, and surrounding areas (Figure 7(c)). In keeping with Bennett and colleagues [40], we identified three main types of sensory papillae and for simplicity refer to them as follows: S1, a sensory cell emanating between the spines with a bulbous base and possessing a prominently projecting cilium; S2, a smooth dome shaped sensory structure lacking a cilium and common inside and around the oral sucker. Six prominent and regularly spaced dome receptors are also found on the rim of the ventral sucker of the 21 DPE parasites cultured with spheroids (Figure 7(f)); S3; these more shielded receptors are spiral or circular with a central pad-shaped structure in the centre (Figure 8).

### Co-culture with spheroids promotes changes in expression and excretion/secretion of parasite cysteine proteases essential for host invasion

We examined the influence of HepG2 spheroid (3D) co-culture on the production and secretion of two developmentally regulated native *F. hepatica* cysteine proteases, cathepsin L3 (FhCL3), which we have shown is a major cysteine protease secreted by NEJ, and FhCL1/2, which are proteases that are associated with migration in the liver by immature parasites and blood feeding by adult parasites in the bile ducts [34,35,43,44].

**Figure 9.**
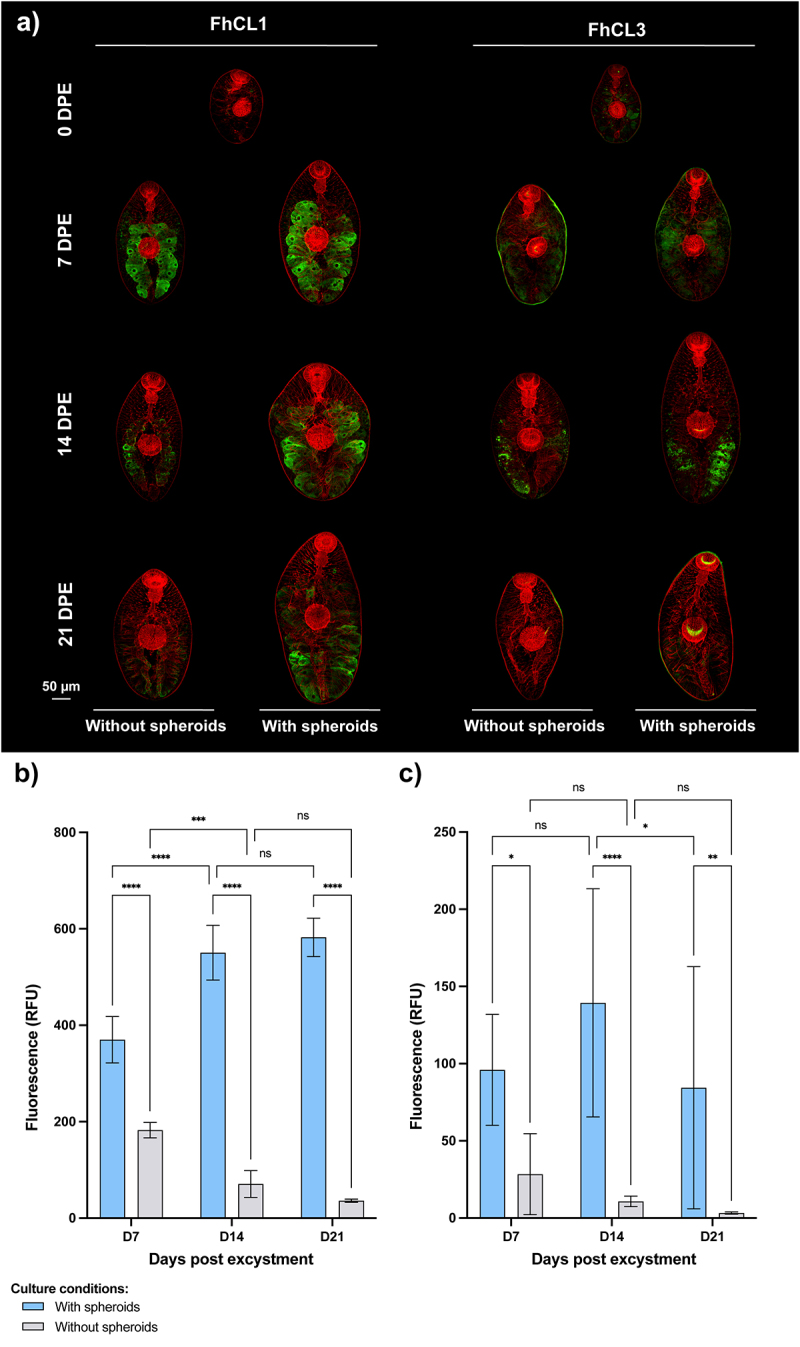
Co-culture of *F. hepatica* NEJ with HepG2 spheroids promotes developmental changes in the expression of the gut cysteine proteases FhCL3 and FhCL1/2. (a) Immuno-localisation of native cysteine proteases, FhCL3 (anti-rFhcl3) and FhCL1 (anti-rFhcl1), in *F. hepatica* parasites at 0 h, 7, 14, and 21 days post-excystment (DPE) with and without HepG2 spheroid co-culture. Immuno-localisation of native cysteine proteases is represented by green fluorescence (FITC staining). Parasite musculature was counter-stained with phalloidin-tritc (actin, red fluorescence). Scale bars: 50 μm. (b) Enzyme activity of native FhCL1 and (C) FhCL3 in cell culture supernatants. Media was collected at 7, 14 and 21 DPE from wells containing parasites cultured with and without HepG2 spheroids. Media from wells containing spheroids only or media alone were used as negative controls (background signal). Activity is expressed as relative fluorescent units (RFU). Significant differences between the two groups (parasites cultured with spheroids vs without spheroids) at 7, 14 and 21 DPE, and significant differences amongst the same group (parasites cultured with spheroids or without spheroids) at different timepoints (7, 14 and 21 DPE) were assessed using a two-way repeated measures ANOVA with Tukey’s *post hoc* test, *p < 0.05, **p < 0.01, ***p < 0.001, ****p < 0.0001.

Scanning laser confocal microscopic images of parasites immunostained with anti-FhCL3 or anti-FhCL1 antibodies are presented in Figure 9. These show that when parasites are co-cultured with spheroids, the expression of FhCL3 decreases over time from 0 DPE to 21 DPE (Figure 9(a)), while the increased expression of FhCL1 is observed from as early as 7 DPE.

FhCL3 hydrolytic activity can be distinguished from FhCL1/2 using enzyme assays with specific fluorogenic peptide substrates, namely, Z-Gly-Pro-Arg-AMC and Z-Phe-Arg-AMC, respectively. Consistent with our immuno-localisation results, supernatants collected from parasite-spheroid co-cultures for these specific enzyme activities contained significantly higher FhCL1/2 enzyme activity compared to cell culture supernatants collected from wells containing parasites alone at 7, 14, and 21 DPE (Figure 9(b)). No activity was detected in wells containing spheroids or media only (Supplementary Data S3). The results of our FhCL3 enzyme activities were less consistent between the two independent experiments, but a significant difference between parasites cultured with and without spheroids was nonetheless apparent at each time point across both experiments (Figure 9(b), Supplementary Data S3).

## Discussion

Infection of humans and animals with the digenean trematode *F. hepatica* begins following the ingestion of vegetation contaminated with infectious encysted metacercaria [18,23]. Microscopic newly excysted juveniles (NEJ, ~100 × 50 μm) emerge from the cysts in the small intestine and traverse the wall of the intestine. Early histological studies show that NEJ find little resistance in penetrating the mucous layer and the single-layered gut endothelium, and they readily breakthrough the surrounding circular and longitudinal muscle layers [31,45–47]. This tissue penetrating process is powered by NEJ glycogen stores and facilitated by the release, *via* the opening of the digestive tract at the oral sucker, of an array of very stable and potent hydrolytic cysteine proteases, cathepsins L and cathepsins B, that are specifically adapted to degrading host tissue interstitial macromolecules such as collagen, laminin, and fibronectin [31,44,48–51]. The drawing actions of the ventral sucker hold and stabilise the parasite within the tissue, while the concerted thrusting mechanical forces exerted by circular, longitudinal, and diagonal muscles drive the parasite forward [31,45,52]. The NEJ respond to a variety of chemical and tactile cues with a repertoire of movement behaviours co-ordinated via a complex muscular and nervous system [45,53,54].

The recent development and characterisation of intestinal organoids derived from rodents and ruminants [4,5] opens wide-ranging possibilities for detailing helminth interactions with this critical host interface *in vitro*. Murine organoids have been used to study the invasion of the intestinal tissue by the early-stage nematode *Trichuris muris* and revealed how they use proteases to degrade the mucus layers before penetration of the epithelial cells, passage into the cell cytoplasm and development of syncytial tunnels [6,10,55]. Livestock organoids derived from ovine and bovine abomasal and illeal tissue permit the short term (<48 h) co-culture of a range of veterinary pathogens including *Teladorsagia circumcincta, Salmonella enterica* serovar Typhimurium, and *Ostertagia ostertagi* [7,8].

While organoids for the investigation of NEJ infection of the intestine are not yet available, an *ex-vivo* model, using sections of the small intestine of rodents into which NEJ are injected before ligation at both ends, has been used to investigate the process of intestinal wall translocation by the early *F. hepatica* stages; parasites traverse and penetrate the intestinal wall into tissue culture medium in similar times, 2–3 h, as this journey takes *in vivo*. Using this technique, we have shown that RNAi-mediated knockdown of parasites cathepsin L and cathepsin B [56] impaired the parasites’ ability to cross the intestinal wall. More recently, Garcia et al. [57] used small opened sections of rat distal jejunum clamped between two chambers holding medium to monitor the passage of the parasite across the intestinal sheet; they validated the method by showing that binding of lectins to the NEJ surface blocks their migration [46].

Over recent decades, many approaches have been taken to develop long-term cultures of juvenile parasites *in vitro* to enable researchers to probe parasite behaviour, development, and surface changes, and to assess their responses to antibodies and anthelmintics [58]. NEJ cultured in a medium supplemented with red blood cells [59] or with chicken serum [59,60] can survive for long periods (up to 29 weeks in the latter case) and exhibit gradual development of internal organs, such as the gut, and cellular changes in the surface tegument. Hanna and Jura [61] reported that *F. gigantica* NEJ cultured with bovine spleen cell monolayers retained their infectivity even after 60 days *in vitro* if injected into the peritoneum of mice. Recently, Gonzales-Miguel and colleagues [48,50] utilised a 2D *in vitro* model employing mouse primary small intestinal epithelial cells (MPSIECs) to analyse the proteomic responses (interactome) of NEJ and demonstrated the release of cathepsin L3 and L4 cysteine proteases in the degradation of extracellular matrix (ECM) proteins by the parasite. We have shown that NEJ cultured in serum-free Roswell Park Memorial Institute (RPMI) medium alter the expression of >8000 transcripts within 24 h [34,35], while Robb et al. [62] recently found that although juvenile fluke maintained in a culture medium containing 50% chicken serum for 21 days do not develop as advanced as *in vivo* (rats) parasites, 86% of the genes they differentially express are shared; *in vivo* parasites exhibit differences in neuronal gene and miRNA expression and neoblast development. The *in vitro* model described herein is, however, the first to support detailed studies of zoonotic helminths interacting with host tissue, permitting long-term (at least 21 days) co-culture without the need for animal material.

Once the NEJ have reached the peritoneal side of the intestine they make their way to the liver, albeit it is not fully clear how this journey is accomplished [58]. Some studies suggest that the parasites are guided by moving along the abdominal wall until they reach the left lobe of the liver, which lies adjacent to where the intestines meet the diaphragm (hence, acute primary infections predominantly affect this lobe) [53,54,58]. Other studies have shown that NEJ injected directly into the peritoneal cavity of mice make it successfully into the liver and do not deviate from other tissues [61]. Although ectopic infections of tissues such as the kidney, lungs or even brain are described in the literature [19,45,63], these are generally rare and suggest that the parasites are guided in their migration by sensing potent attractive molecular cues from the liver tissue. Whatever mechanism(s) NEJ use to reach the liver, by the time they penetrate, the parenchyma their glycogen energy source has been expended and they rely on gaining nutrients from the liver tissue; in the next 2 months of feeding and migration, the parasites will grow approximately 1000-fold (~2 cm × 1 cm) before entering the bile duct where they complete their maturation and become fecund [23,58,64].

Current exploration strives to obstruct the parasite’s migration from the intestine to the liver with new drugs and vaccines since blocking this event would prevent the clinical manifestations associated with acute and chronic fasciolosis (tissue destruction, immunopathology, and haemorrhaging) [31,34,65]. Early intervention is also critical because immunological studies in mice and ruminants have shown that the parasites exert a potent immunomodulatory influence over the host immune system within the first few days of infection, which ultimately leads to a hypo-immune/tolerogenic state in the host that is unable to clear the parasites [66–69]. Accordingly, a detailed molecular comprehension of the parasites’ sojourn to the liver and an understanding of the factors that stimulate their growth and development in this period is imperative. Unfortunately, investigation of these early migratory stages *in vivo* is practically impossible, even using rodent models, due to the minute size of the parasite after excystment and the difficulty in recovering them from host tissue. It follows that the development of *in vitro* systems that replicate these early migratory events would greatly assist the discovery of new anti-parasite interventions. With this goal in mind, we were motivated by recent developments in *in vitro* 3D cell culture to produce spheroids using human hepatoma HepG2 cells that replicate the physiological conditions of solid liver tissue as closely as possible. Furthermore, in comparison to 2D monolayers, liver-specific functions such as albumin, urea, and glucose secretion, and even bile salt transport and the formation of bile canaliculi-like structures, are significantly enhanced in 3D spheroids [70]. Here, we report methods and protocols for the application of these “mini-livers” and provide an evaluation of these by monitoring the temporal expression and regulation of pivotal digestive cysteine proteases, and by probing the changing surface architecture of the developing parasites using scanning electron microscopy (SEM). We show that 3D spheroids can mimic features of *in vivo* parasite–host interactions that spur the growth, development, and morphology of *F. hepatica* NEJ *in vitro*.

Parasites can be maintained with the same HepG2 spheroids for 1–2 weeks without the challenges associated with monolayer cultures, such as cell over-confluence, scraping, or trypsinisation of cells, and the need for frequent passaging. Additionally, the non-adherent plates used in this system simplify day-to-day parasite and spheroid handling, making it easier to process or replate parasites with new spheroids compared to flat-bottom plates. Also, by utilising an easy-to-maintain HepG2 cell line that facilitates straightforward spheroid generation and maintenance alongside *Fasciola hepatica* parasites, we have developed a robust and user-friendly culture system for the investigation of host–parasite interaction that does not require highly specialised expertise.

It was immediately obvious that over a three-week culture period parasite growth and survival was enhanced in the presence of 3D spheroids compared to 2D cultures containing HepG2 monolayers and was even greater compared to cultures lacking cells. In 2D cultures, we observed that parasites tended to interact with clumped cells within the monolayer cultures indicating that they favoured the physical contact with solid tissue. When advanced to a 3D spheroid culture system, an overnight time-lapse video revealed that NEJ exhibited greater mobility compared to the parasites in culture media alone. Furthermore, the parasites physically interacted with and pressed against the solid tissues (tending to orientate tangentially to the spheroids), wandered away and returned, and even penetrated into the channels created between the spheroid triplets using forward-thrusting movements. We interpret these movements and interactions, which have never been recorded previously, as innate behaviours associated with tissue penetration and migration.

The parasites were observed grazing on the outside of the spheroids. However, one of the most striking findings of our study, revealed by fluorescently labelling 3D spheroid cells to examine feeding behaviour in real-time, was the active ingestion and subsequent digestion of HepG2 spheroid cells by the parasites within the first hours of excystment and co-culture. Parasites displayed this feeding behaviour (rhythmic forward and backward movement of the gut) soon after emerging from the cysts, which underscores the importance of host cell ingestion coupled with nutrient uptake from the medium in NEJ survival and development, and the need for the parasite to quickly switch to tissue feeding before their glycogen reserves are depleted. Feeding activity was concomitant with the development of the branching characteristics of the gut, which became particularly obvious when tissue was ingested and clearly seen moving within the caeca. This observed link suggests that the stimulus of feeding and acquisition of nutrients from the host may be an important trigger for inducing parasite gut development, and possibly the subsequent proliferation of neoblasts that drive development in general [34,60,71,72].

The lining of the parasite gut consists of a single layer of columnar epithelial cells that project finger-like lamella into the lumen [58,73]. Granules packed with cathepsin L proteases move from within the cell cytoplasm to the surface of the lamella and exude their contents into the acidic gut lumen. The same epithelial cells are also involved in the absorption of nutrients from the gut lumen from where they are distributed to internal tissues; hence, the epithelial cells of the parasite gut lining undergo a cycle of secretion and adsorption [58,73]. Because the gut is blind-ended (no anus), its contents are regurgitated approximately every 3 hours to allow the parasite to imbibe another meal [58,73,74]. This cyclic process ensures that digestive enzymes and other gut molecules are released into the host tissues where they enable tissue penetration and extracorporeal degradation of host tissues, as well as actuating control or modulation of the host immune system [66]. In *F. hepatica* and other helminth parasites, the excretion/secretion of cysteine proteases plays a pivotal role in early host colonisation, immune evasion, and tissue invasion [75]. Using confocal immunolocalisation, we demonstrated the expression of the major NEJ cysteine proteases, cathepsin L3 and cathepsin L1, in the epithelial cells of the bifurcated parasite gut. Using specific fluorogenic peptide substrates we also biochemically detected the proteases in the medium in which the parasites were maintained and demonstrated that secretion of these enzymes was much enhanced in parasites that were co-cultured with 3D spheroids (in keeping with the observed development of the gut). More relevantly, we observed the developmental switch in the type of protease expressed, which we previously showed is correlated with parasite migration through different tissues [34,39,44,76]; therefore, NEJ in 3D cultures switched expression from the collagenase-digesting cathepsin L3 to the more blood-feeding cathepsin L1/2 proteinase over the 3 weeks of co-culture, providing direct biochemical support for the induction of growth and development of the parasite in response to contact with HepG2 3D spheroids.

SEM was used as a tractable approach to explore the parasite surface for structural and developmental changes associated with their interactions with spheroids in 3D cultures. The most discernible structures on the parasite’s surface are single-pointed spines that project backwards and aid the directional migration of the parasite through tissues. Bennett [40,41] described how the NEJ exhibited a strict arrangement of 60 rings, each with 60 to 70 spines, anterior to the ventral sucker, and showed that their number and size increased as the parasite developed. Indeed, in the present study, we observed a similar arrangement of spines which formed a series of rings around and below the anterior sucker of the NEJ; below the ventral sucker, the spines were less and more randomly dispersed. In contrast to parasites cultured in the medium alone, the size of the surface spines, and the spaces between them, increased in parasites cultured in the presence of spheroids, albeit the general concentric arrangement of the spines remained the same as the parasite grew. The parasites’ spines and tegumental surfaces showed no signs of defective development after 3 weeks of spheroid co-culture.

Another very conspicuous surface feature of developing *F. hepatica* following infection of the host *in vivo* is the expression of a variety of “sensory papillae.” First described by Bennett and Threadgold in 1975 [41], these sensory cells are believed to relay the complex environmental messages associated with the various tissue physiologies to the parasite as it migrates. In agreement with Bennett [40], our studies show that these sensory structures are underdeveloped in the NEJ that immediately emerges from the cyst; we observed that they remained underdeveloped in parasites that were cultured in the medium alone. However, from as early as 7 DPE, the sensory structures become very apparent on the tegument of parasites that were cultured with spheroids. The sensory structures are most numerous and complex at the anterior end of the parasite, particularly around the inner and outer rims of the oral sucker at the tip of the parasite, and in the surrounding areas. We could identify three morphologically distinct types of sensory structures and have maintained the general classification described by Bennett and colleagues [40–42], now referring to them as follows: S1, a sensory cell emanating between the spines with a bulbous base and possessing a prominently projecting cilium which suggests a function in mechanoreception; S2, a smooth dome shaped sensory structure lacking a cilium that is particularly common inside and around the oral sucker. Six of these are very prominent and regularly spaced on the rim of the ventral sucker of the 21 DPE parasites cultured with spheroids, suggesting that this type of sensory structure is important for searching and probing the substrate and environment, and thus facilitating attachment or anchoring of the parasite in the host tissue. The head region of the juvenile and migrating flukes can move independently of the posterior part of the parasite, and movement through tissue is achieved by a coordination of alternate attachment and release of oral and ventral sucker [40,52,58]. Given that S1 and S2 receptors are most abundant on the oral and ventral suckers, which are highly muscular, these likely facilitate and coordinate the exploration of the host tissue, migrating, tissue penetration, and feeding; S3; these more shielded receptors are spiral or circular with a central pad-shaped structure in the centre. They are more randomly distributed in the anterior part of the parasite and, as suggested by Bennett, they could complete the *F. hepatica* set of sensory structures by being “taste” buds [40] that may detect and relay messages from chemical cues released from various tissues.

While the functions attributed to each of these sensory structures are solely based on their morphology and location, their elaborate expression during co-culture with HepG2 spheroids suggests that the physically tactile interactions between parasites and spheroids (revealed here in real-time, supplementary Video 3) are essential to stimulating the development of sensory perceptive mechanisms and the expression of sensory structures on the surface tegument. Closer investigation of these various sensory papillae, particularly regarding their relationship with the parasite neural system and information transmission to the central ganglion, will reveal their true functions. Furthermore, the characterisation of each at a molecular level is essential as sensory cells represent an exceptional prospect for developing vaccines or drugs that impede parasite host-sensing, infection, migration, feeding, and infection. Towards this goal, McVeigh et al. [77] identified 147 G-protein coupled receptors in the transcriptome of *F. hepatica* [34,35,78], many of which are more highly expressed in the NEJ and early liver-stage parasites and await functional characterisation.

Foodborne zoonotic trematodes infect >300 million people across the globe, primarily in rural low-income regions. Control of these diseases relies on very few compounds; the most notable frontline drugs are praziquantel for schistosomiasis, opisthorchiasis and clonorchiasis, and triclabendazole for fasciolosis. This severe dearth of treatments underscores the urgent need to discover new interventions, both drugs and vaccines. *In vitro* cultivation methods play a central role in the efforts to understand parasite biology to uncover new potential vaccine targets and, indeed, as a simple early means to test and screen new anti-parasitics. Accordingly, there is a need to have these *in vitro* cultures resemble the *in vivo* setting as closely as possible, at least for the part of the life cycle that represents a targetable prospect in the relevant host [3,31,65]. In this report, we describe the use of HepG2 3D spheroids to recreate solid tissues that mimic the environment and physiology of liver tissue and form a suitable substrate for *F. hepatica* NEJ to interact. It must be noted, however, that HepG2 cells do not completely recapitulate the complexity and normal function of liver tissue [79]. Nevertheless, our observations suggest that the interactions and physical contacts between spheroids and parasites are critical to stimulating development, particularly of the tegumental spines and various sensory papillae, and that the feeding behaviour is essential to stimulating growth and development in the host. It will be of interest to explore different immortalised liver cell lines, such as HepaRG cells, either homotypically or as HepG2/HepaRG heterotypic spheroids (and indeed cells derived from other tissues), to assess whether these offer additional enhancements to developing NEJ.

These 3D spheroid culture methods add greatly to the varied *in vitro* culture systems described above that are already available to probe this parasite’s development and complement emerging advancements in 3D organoid cultures. This is the first report on the use of spheroids to investigate worm–host interactions but, undoubtedly, other parasite systems may benefit from a similar approach. Major advantages associated with the use of 3D spheroids include their relative ease of preparation and their suitability for upscale production of medium-to-high throughput screening tests, such as antibodies, drugs, and interference RNAs, for both vaccine and anthelmintic discovery projects. In the case of drug screening, the anti-parasitic activity and toxicity to solid tissue of compounds could be scrutinised simultaneously. Moreover, compared to 2D cultures, 3D spheroid models exhibit increased cytochrome P450 (CYP) enzyme expression, which is critical for drug metabolism, including that of triclabendazole, the only flukicide effective against juvenile flukes [17,70,80]. For our own purpose, we will exploit this methodology for the *in vitro* short-listing of vaccine candidates before these are taken forward into large animal vaccine trials in accord with the 3 R principles of reduction, refinement, and even replacement of animal models in experimentation [17,32,65].

## Supplementary Material

Captions for the supplementary videos.docx

## Data Availability

The data that support the findings of this study and the supplementary files are openly available in Figshare at https://doi.org/10.6084/m9.figshare.26968969.v2.
