# Supplementary material for: In vitro co-culture of Fasciola hepatica newly excysted juveniles (NEJs) with 3D HepG2 spheroids permits novel investigation of host–parasite interactions
Source: Virulence. 2025 Mar 25;16(1):2482159. doi: 10.1080/21505594.2025.2482159 (PMC11938319; doi:10.1080/21505594.2025.2482159)
Supplement: Captions for the supplementary videos.docx [file KVIR_A_2482159_SM8196.docx]

**Captions for the supplementary videos**

**Supplementary video 1.** 14-day *Fasciola hepatica* parasite cultured with a monolayer of HepG2 cells *in vitro*. 2x speed. Scale bar: 100 μm.

**Supplementary video 2.** A timelapse of 0-hour *Fasciola hepatica* newly excysted juveniles cultured without HepG2 spheroids *in vitro*. Images were taken every 30 seconds for 13 hours. Speed: 10 frames per second (fps), scale bar: 200 μm.

**Supplementary video 3.** A timelapse of 0-hour *Fasciola hepatica* newly excysted juveniles cultured with HepG2 spheroids *in vitro*. Images were taken every 30 seconds for 13 hours. Speed: 10 frames per second (fps), scale bar: 200 μm.

**Supplementary video 4.** A timelapse of 21-hour *Fasciola hepatica* newly excysted juveniles cultured with HepG2 spheroids labelled with a fluorescent PKH67 green cell membrane dye. Images were taken every 60 seconds for 21 hours. Speed: 10 frames per second (fps), scale bar: 200 μm.
